# Effect of Chewing Gum on Oral Mucositis in Children Undergoing Chemotherapy: A Randomized Controlled Study

**Published:** 2016-03-15

**Authors:** A Eghbali, B Taherkhanchi, B Bagheri, B Sadeghi Sedeh

**Affiliations:** 1 **Department of Pediatric Hematology and Oncology, School of Medicine, Arak University of Medical Sciences, Arak, ran**; 2 **Department of Pediatrics, School of Medicine, Semnan University of Medical Sciences, Semnan, Iran.**; 3 **Cancer Research Center and Department of Pharmacology, School of Medicine, Semnan University of Medical Sciences, Semnan, Iran.**; 4 **Department of Community Medicine, School of Medicine, Arak University of Medical Sciences, Arak, Iran.**

**Keywords:** Chemotherapy, Children, Gum, Mucositis

## Abstract

**Background:**

Oral mucositis is an adverse effect of chemotherapy. Type of chemotherapy regimen is the most important factor causing mucositis. Oral mucositis is usually associated with transient decrease in saliva production. The goal was to study effects of gum consumption on oral mucositis in children undergoing chemotherapy.

**Materials and Method:**

This randomized controlled trial was done in Amir Kabir Hospital, Arak, Iran. 130 children 5 to 15 years of age were studied. Control group was composed of 65 children who received mucotoxic drugs. Test group was made up of 65 patients received similar drugs in addition to sugar free gums. Patients consumed 6 pieces of gums per day for 15 days. A standardized follow up form and World Health Organization (WHO) grading system for oral mucositis were used for evaluation of patients during 15 days of treatment.

**Results:**

Severe oral mucositis occurred in 30 (46%) of 65 patients in the test group and in 26 of 65 (40%) patients in the control group. Difference was not statistically significant (P > 0.05). Rate of mild to moderate mucoitis (grade 1 and 2) was significantly lower in patients who used gums (15 % vs. 35%, P < 0.05).

**Conclusion:**

Our study showed that stimulation of saliva flow by chewing gum could decrease mild to moderate inflammatory injuries of the oral mucosa during chemotherapy. However, it was not effective to subside severe mucositis.

## Introduction

Oral mucositis is one of the most common untoward effects of cancer chemotherapy and radiotherapy (1). Oral mucositis is an inflammatory condition, which is usually severe and painful. Types of medication and patients’ factors are determining factors for the severity of the disease (2,3). Evidence from several lines of studies indicates that innate and adaptive immunity are involved in initiation and progression of mucositis. Pro-inflammatory cytokines like IL-1β and TNF-α are abundantly found in oral lesions related to cancer chemotherapy. These cytokines can worsen the inflammatory status by increasing the permeability of the oral tissues and causes further accumulation of immune cells and cytotoxic agents (4-6). In addition, it is suggested that cytotoxic agents have some direct effects on oral epithelium which can lead to damage of acinar ductal cells of the salivary glands and hyposalivation may happen (7). Naturally, saliva is composed of antimicrobial and mucosal protective compounds that are vital for the oral health (8,9). Therefore, any decrease in saliva can predispose the oral cavity to the damaging effects of cancer chemotherapy. During recent years, several guidelines have been applied to prevent mucositis (10,11). Currently, the most effective means of preventing mucositis is through good oral hygiene (12). It is thought that stimulation in salivary production may decrease the severity of mucositis. Chewing gum can stimulate the salivary production up to 10-fold (13). Importantly, sugar-free gums are inexpensive and available. The purpose of the present was to study effects of gum on oral mucositis in children undergoing chemotherapy. This study was implemented to find whether gum consumption could subside the severity of mucositis.

## Materials and Method

This single center, randomized, controlled trial was conducted in Amir Kabir Hospital, Arak, Iran, between April 2014 and June 2015. The sample size was calculated with a type I error of 5%, a statistical power of 80%, and a standardized effect size of 0.65. A total of 130 children 5 to 15 years of age who were receiving same mucotoxic drugs were randomized 1 : 1 to 2 groups: control group and test group. Control group included 65 patients who were given a standard mouth rinse composed of nystatin,diphenhydramine, and aluminum Mgs, 3 times a day. Test group included 65 patients who were administered the mentioned mouth rinse and gum. Chewing gums were sugar-free (White^®^, Minoo, Tehran, Iran). Patients consumed 6 pieces per day for 15 days. Chewing lasted about 30 minutes. Each child participated for 1 chemotherapy course. The exclusion criteria were patients older than 15 years old or younger than 5 years old, patients were not on stomatotoxic agents, patients with diabetes, patients with cardiovascular diseases, patients suffered from mental disorders, and patients with oral lesions caused by any reason. Medications, sex, age, and previous medical history were obtained through questionnaire and medical records of the patients. The local Ethics Committee approved the study and informed consent was obtained from parents of participants. 

**Evaluation of Patients**

During chemotherapy, physicians evaluated patients. Severity of mucositis, health of oral cavity and general status of the patients were adequately assessed and classified according to WHO scoring system (Table I). After termination of chemotherapy, routine evaluations were done every 3 days for a total of 15 days.

**Statistical analysis**

Data are reported as mean ± SD or numbers (percentage). Data distribution was checked by Kolmogorov–Smirnov test. Means were compared by Wilcoxon signed rank test. Differences at the level of P < 0.05 were considered statistically significant. All analyses were performed using SPSS 16.0.

## Results

**Clinical characteristics**

Table II shows clinical characteristics of study subjects. There were no significant differences in demographics or clinicopathological features between the 2 groups (P > 0.05). 

**Effect of chewing gum**

**Figure 1:**
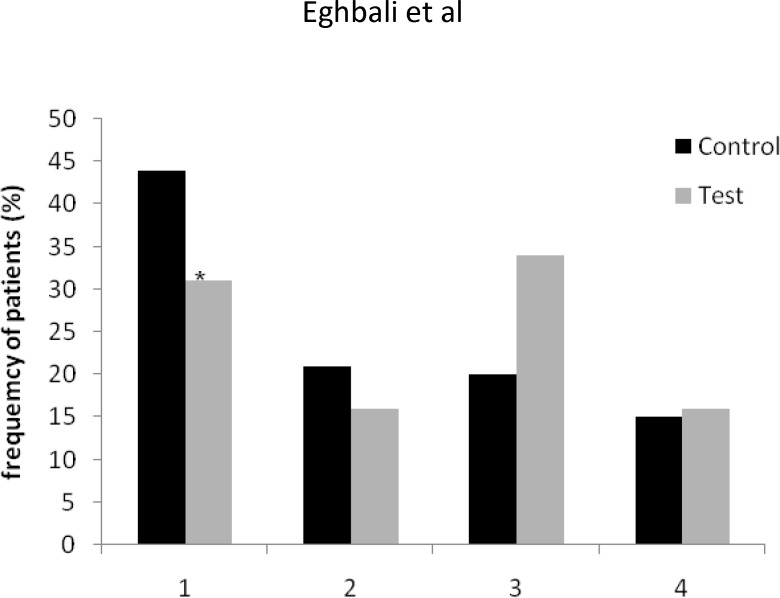
Effect of gum consumption on different stages of oral mucositis.^*^ P < 0.05

As assessed by WHO scoring system, a significant reduction was seen in incidence of grade 1 mucositis, as compared to control group (44% vs 30%, P < 0.05; Fig. 1). In addition, incidence of grade 2 mucositis was lower in test group as compared to control group (20% vs 15%, Fig.1), however, this difference failed to reach at a significant level (P>0.05). In advanced grades (3 and 4), no positive effect of chewing gum was seen. As shown in Fig1, grade 3 mucositis was more frequent in test group.

**Table I:** *WHO scoring system of mucositis*

**Description**	**Grade**
**No mucositis present**	0
**Irritation of oral mucosa, with pain; no overt ulceration**	1
**Sores evident in oral mucosa; patients still able to swallow solid food**	2
**Patient experience extreme sensitivity when swallowing solid food**	3
**Patient unable to swallow**	4

**Table II:** *Clinical characteristics of study patients*

**Characteristic **	**Control (N=65)**	**Test (N=65)**
Age (yr)	8 ± 2.1	9 ± 2.7
Male	39 (60)	27 (41.5)
Malignancy		
AML	14 (21.5)	19 (29.2)
ALL	32 (49.2)	27 (41.5)
T lymphoblastic leukemia	10 (15.3)	13 (20)
Osteosarcoma	5 (7.6)	3 (4.6)
Rhabdomyosarcoma	4 (6.1)	1 (1.5)
Ewing	1 (1.5)	2 (3)
Neutrophil count	2921 ± 1254	2856± 1237

Data are shown in number, percentage (%) and mean ± SD

## Discussion

This randomized and controlled study showed that gum consumption could decrease the severity of oral mucositis in children undergoing chemotherapy. Chemotherapy-induced oral mucositis is very common and usually hard to control. Oral mucositis is often a hurdle to continue the schedule of treatment and can be a cause of failure in treatment. Such unexpected intervals in treatment of malignancies, may eventually lead to poor outcome of treatment (14). Thus, management of mucositis and other common side effects of cancer therapy are of great value. It is suggested to use drug combinations, which are less likely to cause mucositis especially in children who have concomitant dental or oral diseases (15). Poor oral hygiene is very important risk factor, which is usually linked to the severity of mucositis. It has been demonstrated that children who have healthier status of oral mucosa are more resistant to oral lesions of chemotherapy (15-17). The results of current study showed that gum consumption had no significant effect on advanced mucositis; grade 3 and 4. Importantly, in grade 3, patients who had used gum were more susceptible to mucositis. It is obscure that how gum could reduce the frequency of low-grade mucositis, grade 1 and 2. However, no significant difference was seen between two groups at grade 4. It may be partially explained by individual differences in patients and drugs. As noted earlier, it is very difficult to control advanced mucositis. Cumulative evidence has shown that stimulation of salivary flow can reduce the severity of mucositis (17,18). Saliva is composed of different compounds, which can protect the mucosa. Pathophysiologically, saliva is one of mechanisms involved in preservation of oral mucosal integrity (13). Somatotoxic drugs are able to directly affect the fundamental structures of oral mucosa; hence, salivary stimulation is not strong enough and for sure not the only defensive mechanism against injuries of these drugs (19,20). Adjustment of oral PH by saliva can be new field of research. It should be further investigated that which PH has maximal efficacy against injuries of drugs. Taken together, it is suggested that all children undergoing chemotherapy use chewing gum for reduction of oral mucositis, especially low grade mucositis. More studies are needed to gain fuller understanding about the other mechanism involved in progression of oral mucositis. New targets will be new area of research.

## Conclusion

This trial shows that using chewing gum for 15 days is effective to prevent moderate mucositis in children, albeit it has no significant effects on advanced mucositis. 
